# Peut-on réaliser une analgésie péridurale après un blood patch récent?

**DOI:** 10.11604/pamj.2015.22.10.6703

**Published:** 2015-09-07

**Authors:** Khalid Chkoura, Hicham Kechna, Jaouad Loutid, Omar Ouzad, Moulay Ahmed Hachimi, Sidi Mohamed Hannafi

**Affiliations:** 1 Pôle d'Anesthésie Réanimation et Urgences, Hôpital Militaire d'Instruction Moulay Ismail, Meknès, Maroc

**Keywords:** Syndrome post ponction lombaire, péridurale, blood patch, Post lumbar puncture syndrome, epidural, blood patch

## Abstract

Le blood patch constitue le traitement de référence du syndrome post ponction lombaire. Son efficacité a été démontrée par plusieurs essais randomisés comparant le BPE aux mesures conservatrices et au placebo. Il consiste en l'injection d'une quantité de sang autologue dans l'espace péridurale afin d'obturer la brèche dure-mérienne. La réalisation d'une analgésie péridurale après antécédent de blood patch est possible, malgré la persistance de questions sur les délais de retour à la normal de l'espace péridurale. Nous rapportons un cas où une analgésie péridurale a été réalisée avec succès trois semaines après un blood patch.

## Introduction

Depuis sa description il y'a plus d'un demi siècle, le Blood Patch (BP) constitue le traitement de référence du syndrome post ponction lombaire (SPPL). Ce syndrome intéresse une population jeune qui peut avoir recours à d'autres anesthésies péri médullaires. Chez de telles personnes, des questions peuvent se poser et plus particulièrement celle concernant le moment à partir duquel le praticien peut leurs proposer une nouvelle expérience d'anesthésie péri médullaire? Nous rapportons le cas d'une jeune femme de 46 ans qui a bénéficié d'un blood patch à la suite d'une brèche dure mérienne au décours d'une péridurale. Trois semaines après, une anesthésie péridurale a été pratiquée et les suites ont été favorables.

## Patient et observation

Il s'agit d'une patiente âgée de 46 ans, sans antécédents pathologiques notables et de corpulence normale, opérée à deux reprises pour une résection d'une tumeur recto sigmoïdienne sous anesthésie générale associée à une analgésie péridurale lombaire. Dans les suites opératoires de la première intervention, la patiente a présenté des céphalées positionnelles associées à des cervicalgies, des bourdonnements d'oreilles et des nausées sans fièvre entrant dans le cadre d'un syndrome post ponction lombaire qui a été rattaché à une brèche dure mérienne provoquée accidentellement lors de la réalisation de la péridurale. Après échec du traitement conservateur, le recours à un blood patch au cinquième jour du post opératoire a été nécessaire. Vingt millilitre de sang autologue a été injectée dans l'espace péridural. L'efficacité était rapide avec un soulagement de la patiente facilitant sa déambulation. Après des séances de radiothérapie, la patiente est admise au bloc pour un traitement radical de la tumeur trois semaines après la première intervention. Le protocole anesthésique proposé était de nouveau une anesthésie générale associée à une analgésie péridurale lombaire. La réalisation de la péridurale n'a pas présenté de difficultés particulières et les suites étaient favorables.

## Discussion

Le SPPL peut compliquer tous les types de ponctions durales, qu'il s'agisse d'une ponction lombaire, d'une rachianesthésie (RA) ou d'un accident d'anesthésie péridurale (qui normalement ne perce pas la dure mère). Sa fréquence est très variable de 1 à 70% selon les études. Il est plus fréquent chez l'adulte jeune [1], de sexe féminin [1, 2], mince [3], ayant des antécédents de céphalées ou ayant eu un précédent SPPL. Le Blood Patch (BP) décrits par Gormley en 1960 [4], consiste en l'injection d'une quantité de sang autologue dans l'espace péridural constitue le traitement de référence des céphalées compliquant les brèches dure-mériennes en cas d’échec du traitement conservateur. Son objectif est de restaurer le régime de pression habituel du LCR en obturant la brèche. Expérimentalement, le sang injecté accélère la cicatrisation de la dure-mère expliquant la rareté des SPPL après brèche durale au cours des actes neurochirurgicaux. Par ailleurs, le LCR jouerait un rôle activateur de la coagulation sur le sang injecté [5]. Bien que le sang soit rapidement résorbé (dès trois heures après la réalisation du BP), le clou plaquettaire reste présent au moins sept jours après [2, 6]. En 1975, Abouleish E. et al [7] ont publié une étude au cours de laquelle ils ont mentionné que la réalisation d'une anesthésie péridurale ou rachidienne chez deux patientes ayant bénéficié d'un blood patch moins de 380 jours auparavant, n'avaient présenté aucun problème.

En 2001, Nouri M. et al [8], rapportent le cas d'une rachianesthésie pratiquée chez une patiente ayant eu un blood patch deux années auparavant. Le déroulement de la rachianesthésie et ses suites ont été normales. Le cas de cette patiente et l'interrogation des auteurs illustrent des questions fondamentales: comment évolue l'anatomie de l'espace péridural et de la dure-mère après un blood patch et peut-on pratiquer ultérieurement une anesthésie péri médullaire avec succès et sans risque majoré de nouvelle fuite de LCR et de céphalée? Efficace et sûr, le blood patch ne grève-t-il pas l'avenir du patient pour la pratique de futures anesthésies péri médullaires? Il est bien connu qu'en répétant les rachianesthésies on obtient des blocs chaque fois similaires [9]. Il est également admis qu'après une brèche dure-mérienne, avec ou sans blood patch, on peut pratiquer des anesthésies péridurales sans risque majoré d’échec ou de nouvelle brèche dure-mérienne. Ceci est en faveur au fait qu'après une brèche dure-mérienne traitée par un blood patch «l'anatomie fonctionnelle» locale n'est pas modifiée de façon significative. Devant l'absence d’études sur le temps nécessaire à la normalisation de l'espace péridural après réalisation d'un blood patch, il semble prudent, mais peut-être pas indispensable, de pratiquer la rachianesthésie un espace au-dessus ou en dessous de celui de la brèche dure-mérienne précédente. Enfin à l'heure actuelle, les praticiens référents sont rassurants sur le devenir des patients ayant eu un blood patch et considèrent, jusqu’à preuve du contraire, qu'une vie normale est possible après un blood patch [10]. Pour le cas de notre patiente, les suites favorables après la réalisation d'une péridurale trois semaines après un blood patch laisse penser qu'une anesthésie péri médullaire est possible après un blood patch réalisé au décours du même mois.

## Conclusion

Après une brèche dure-mérienne, la réalisation d'une anesthésie péri médullaire est possible sans risque majoré d’échec ou de nouvelle brèche dure-mérienne.
